# When do you scratch that itch? The relative impact of different factors on scratching depends on the selection of time scale and timing

**DOI:** 10.1098/rsos.170106

**Published:** 2017-04-05

**Authors:** Ivan Norscia, Elisabetta Palagi

**Affiliations:** Museo di Storia Naturale, Università di Pisa, Via Roma 79, Calci, Pisa 56011, Italy

In their interesting article, Duboscq *et al*. [1] used behavioural data to examine equitably a range of hypotheses on the factors that may cause scratching variations in their wild group of Japanese macaque (*Macaca fuscata fuscata*). They concluded that the animals scratched ‘primarily because of an immune/stimulus itch, possibly triggered by ectoparasite bites/movements’ [1, pp. 1–2] but did not exclude that other factors may have a secondary role. In this Commentary we explain why, in our opinion, the definition of the primary and secondary role of different factors, even within the same group and period, is not absolute but rather time scale and timing dependent. Among other types, stressors can be *acute* if they have rapid onset and/or short course or *chronic* if they extend over a prolonged period of time. The response to different types of stressors (e.g. threatening incidents, uncomfortable weather, ectoparasites) also reflects this difference [2,3].

The authors put forth different hypotheses on self-directed behaviours related to the effect that parasitological (presence of ectoparasites), environmental (temperature and humidity/rainfall) and social factors (e.g. aggression, proximity with higher ranking individuals, etc.) have in affecting the rates of self-directed behaviours, including scratching. Such hypotheses are presented as alternative in the first part of the Introduction [1, p. 2] and in the Method section [1, p. 8], but the authors themselves define them as ‘mutually non-exclusive’ [1, p. 3] and test them via different statistical models in which the three groups of factors are included either separately or together. Other than not being alternative, the hypotheses can no longer be considered as such with the present level of knowledge. As Duboscq *et al*. [1] also explain, in either the Introduction [1, p. 2] or Discussion [1, p. 11], that ectoparasites can directly trigger ‘immune’ itch (via immune response) [4,5] whereas temperature and stress can cause non-immune itch. In particular, the environmental temperature, by possibly causing skin temperature changes, can activate C neural sensory fibres, involved in the sensation of itch [6]. In humans and other mammals, stress hormones are related to skin inflammatory processes and can also lead to dermatitis and itching [7]. Whatever the triggering stressor (or stressors) may be, itching activates the scratch reflex. Moreover, as briefly pointed out by Duboscq *et al*. [1], the strong linkage between stress-induced hormones and self-directed behaviours has been demonstrated in monkeys via pharmacological trials [8]. In macaques, self-scratching is sensitive to anxiolytic and anxiogenic substances [9]. In primates, spanning lemurs and apes, the scratching rates measured in the minutes following a stressful event can be reliably used to measure anxiety and are sensitive to different kinds of homeostasis perturbation (e.g. predation attempt, aggression, proximity of dominant individuals) [10–15]. Hence, the association between self-directed behaviours, and particularly scratching, with social, environmental and parasitological factors can be considered as more than just a hypothesis.

Once established that the different factors are not alternative and that their relationship with scratching has been demonstrated, it is worth focusing on the role that each factor can have in relation to the time scale. We will particularly focus on scratching. The framework of Duboscq *et al.* [1] could benefit from considering not *if* but *when* a certain factor can be more effective than another in producing scratching. Stressors can be additive but they operate in the short- or long-term depending on whether they are transient (acute) or prolonged (chronic) [3]. Thus, if concurrently present they can have a combined action on the behavioural response. However, determining the relative effect of each factor on scratching may not be possible all at once by using a single methodological approach because the effect of multiple stressors cannot be quantified using incomparable time scales. Normalizing the behavioural bouts over the observation minutes does not solve the problem because, as we explain below, timing (when) other than time scale (how long) is fundamental.

In the case of the Duboscq *et al*. study group, the scratching due to long-lasting ectoparasite load (the authors use monthly average values for this variable) can be the primary component of the overall baseline scratching levels. However, this does not exclude the possibility that scratching can significantly increase—compared with baseline—in response to a transient stressor, such as a conflict. In the short period around the event, the aggression can be primarily responsible for scratching variation but only a sequential, temporal analysis can unveil this aspect [16]. The scratching reaction (activated by itching) to an episodic stressful event is a matter of minutes. For this reason, previous studies on post-conflict behaviour considered the exact time of the aggressive event (*t*_0_) and the tight time window (*t*_0_ − *t*_1_) immediately following the aggressive event in order to detect the variation of scratching levels [17]. The variation observed between time *t*_0_ and *t*_1_ cannot be linked to parasite load if the load is not significantly different between *t*_0_ and *t*_1_. There is no reason to believe that, in the absence of any other additional perturbing factor, the ectoparasite load varies significantly in the minutes immediately preceding and following the stressful event. Of course, an extra control measure can be taken in this respect if deemed necessary. It may be questioned that in the short term a change in the parasite activity (e.g. in response to temperature, humidity or even solar radiation [18–22]), and not in the load, could possibly cause an increase in the scratching levels. However, this aspect was not tested in Duboscq *et al*. [1]. Another type of study design, involving the detection of parasite positions, movements and temporal analyses, would be necessary to evaluate this aspect.

In conclusion, the relative weight of the different factors affecting scratching can change depending on the time scale and timing selected. The article by Duboscq *et al*. [1] is valuable in explaining the chronic cause of scratching in their study group and site. However, other types of stressors, such as severe conflicts, can still have a primary, and not a secondary, effect on scratching when acute stressors come into play. We thank the authors because through their work they have opened new interesting scenarios on how to investigate the scratching responses related to stressors that operate under different time frames.
